# A Scheme for Quantum Teleportation and Remote Quantum State Preparation of IoT Multiple Devices

**DOI:** 10.3390/s23208475

**Published:** 2023-10-15

**Authors:** You Fu, Dongfen Li, Xiaoyu Hua, Yangyang Jiang, Yonghao Zhu, Jie Zhou, Xiaolong Yang, Yuqiao Tan

**Affiliations:** College of Computer Science and Cyber Security (Oxford Brookes College), Chengdu University of Technology, Chengdu 610059, China; 2022050888@stu.cdut.edu.cn (X.H.); 2022050894@stu.cdut.edu.cn (Y.J.); 2022050893@stu.cdut.edu.cn (Y.Z.); 2021020861@stu.cdut.edu.cn (J.Z.); 2021050831@stu.cdut.edu.cn (X.Y.); 2021020897@stu.cdut.edu.cn (Y.T.)

**Keywords:** IoT security, quantum communication, controlled quantum teleportation, remote state preparation, multi-device communication

## Abstract

With the continuous development of the Internet of Things (IoT) technology, the industry’s awareness of the security of the IoT is also increasing, and the adoption of quantum communication technology can significantly improve the communication security of various devices in the IoT. This paper proposes a scheme of controlled remote quantum state preparation and quantum teleportation based on multiple communication parties, and a nine-qubit entanglement channel is used to achieve secure communication of multiple devices in the IoT. The channel preparation, measurement operation, and unitary operation of the scheme were successfully simulated on the IBM Quantum platform, and the entanglement degree and reliability of the channel were verified through 8192 shots. The scheme’s application in the IoT was analyzed, and the steps and examples of the scheme in the secure communication of multiple devices in the IoT are discussed. By simulating two different attack modes, the effect of the attack on the communication scheme in the IoT was deduced, and the scheme’s high security and anti-interference ability was analyzed. Compared with other schemes from the two aspects of principle and transmission efficiency, it is highlighted that the advantages of the proposed scheme are that it overcomes the single fixed one-way or two-way transmission protocol form of quantum teleportation in the past and can realize quantum communication with multiple devices, ensuring both security and transmission efficiency.

## 1. Introduction

The IoT refers to various devices and objects connected via the Internet, enabling them to communicate and share information. With the continuous progress of technology, the IoT has become an essential part of modern society, and its application scope covers various fields such as home, industry, medical treatment, and transportation. However, the rapid development of the IoT has also brought a series of security issues. Data transmission in the IoT often needs to be carried out in different network environments, including wireless networks, cloud servers, and so on. This increases the risk of the data being eavesdropped, tampered with, or falsified in transit. Traditional encryption methods may not provide adequate protection, as the rise of quantum computing could threaten current encryption algorithms [1,2,3].

Quantum mechanics has brought new possibilities to the development of the IoT, and so far, various quantum communication and quantum computing technologies have emerged. These include quantum authentication technology [4,5,6], quantum neural networks [7,8], and quantum signature technology [9,10], which greatly enhance computational efficiency and security. Quantum authentication technology aims to verify quantum channels’ security, ensuring communication integrity and confidentiality. It can be used to verify the presence of potential eavesdroppers or tampering between communication parties. However, quantum authentication technology cannot provide absolute confidentiality as it still relies on classical communication for verification and key distribution. Quantum signature technology is used to ensure the non-repudiation of digital signatures, meaning that signers cannot deny the authenticity of their signatures. Although quantum signature technology can provide a certain level of security, it still relies on classical communication and computation for signature verification and identity authentication.

In contrast, Quantum Teleportation (QT) and Remote Quantum State Preparation (RSP) offer a higher level of security. These protocols utilize the properties of quantum entanglement to achieve secure transmission of information at the quantum level. The security of QT and RSP is based on the protection of quantum entangled states and the non-repudiation of quantum states during transmission. Due to the characteristics of quantum entanglement, even if an eavesdropper intercepts the transmitted quantum states, it cannot copy or steal the information contained within, thereby ensuring the security of the information. The development of QT has been rapid, starting with the concept proposed by Bennett et al. [11] in 1993, and now, there are many improved and novel QT schemes and other quantum communication protocols based on it, for example Quantum Information Splitting (QIS), Quantum Controlled Teleportation (QCT), Bidirectional Quantum Teleportation (BQT), Asymmetric Bidirectional Quantum Teleportation (ABQT), and RSP, which differs from the principles of QT. QCT introduces a third-party supervisor in addition to the communicating parties. Completing the communication requires the supervisor’s consent to reconstruct the quantum state sent by the sender.

Afterward, many QCT schemes have also been developed [12,13,14,15,16]. In 2020, Chen et al. [12] proposed a scheme using a seven-qubit entangled state as the quantum channel to transmit Bell states. They considered the scheme’s effectiveness and improved its security by incorporating decoy states. In 2021, Zhou et al. [17] implemented a bidirectional quantum teleportation scheme using a six-qubit quantum channel for quantum state transmission, providing valuable insights for enhancing efficiency. In 2022, Mengting Wang and Hai-Sheng Li [18] achieved bidirectional controlled quantum teleportation, contributing to the ongoing development of bidirectional quantum teleportation protocols [17,18,19,20,21,22]. Asymmetric bidirectional quantum teleportation refers to the scenario where the quantum states transmitted by both parties can be different, allowing for a greater variety of quantum state transmissions. In 2022, Kazemikhah et al. [23] proposed a scheme using an eight-qubit cluster state as the quantum channel for asymmetric bidirectional quantum state transfer. This ABQT scheme has also become an area of interest for researchers [23,24,25]. In addition, there are various types of quantum teleportation, including quantum information splitting [26,27], cyclic quantum teleportation [28,29,30], and probabilistic quantum teleportation [31,32,33]. On the other hand, RSP differs from the principles of quantum teleportation. In RSP, the sender, Alice, knows the quantum state being transmitted, and it is prepared remotely using entangled quantum channels. The concept of remote state preparation was introduced by Bennett et al. [34], adding another consideration to the field of quantum communication. Similar to QT, RSP also encompasses controlled RSP [35,36], bidirectional RSP [37], and various schemes for preparing different types of quantum states.

QT and RSP are also developing, but most current research focuses on one-way or two-way communication. In this paper, a scheme is proposed to realize secure communication between multiple devices in the IoT by using remote quantum state preparation and quantum teleportation. Experiments verified the scheme’s feasibility, and quantum computers were used to simulate quantum circuits and measurement results. This technology enables secure communication between multiple devices in the IoT. Compared with other schemes, the advantage of this scheme is that it overcomes the single fixed one-way or two-way transmission protocol form in the past quantum teleportation, realizes the quantum communication of multiple devices in the IoT, and ensures the consideration of security and transmission efficiency.

The structure of this paper is as follows. The Section 2 introduces the theoretical derivation of the scheme and describes the theoretical construction of the channel and the specific details of the transmission steps. The Section 3 introduces the experimental verification of the scheme, which mainly realizes the specific channel-construction process, the transmission process, and the final quantum-state-reconstruction process through the IBM Quantum platform based on a theoretical derivation. The Section 4 mainly introduces the technology and corresponding steps needed in the practical implementation of this scheme. The Section 5 introduces the security analysis of the scheme in the IoT. It analyzes the security of the scheme by simulating two different attacks and deducing the impact of attacks on the communication scheme. In the Section 6, we make some comparisons with other schemes and briefly analyze the scheme principle and transmission efficiency. In the last section, the thesis and the scheme are summarized.

## 2. Specific Scheme

The scheme aims to achieve controlled RSP and QT. This part mainly expounds upon the scheme from the theoretical part and separately studies the channel-preparation and transmission protocol.

### 2.1. Controlled Quantum Communication Protocol Based on Multiple Parties

Assume that there are five parties in the process of RSP and QT: Alice, Bob, Charlie, David. and Eve. As the sender, Alice remotely prepares quantum states from Bob and Charlie and sends quantum states to David through teleportation. Eve acts as a supervisor and controls the entire transmission outcome.

### 2.2. The Process of Channel Preparation

We prepare the channel with nine qubits, three H gates, and six CNOT operations. First, the nine qubits have an initial state of |0〉, and then, the Hadamard gate and CNOT operations are performed on them to prepare the entangled quantum channel. The entire channel preparation process is as follows:

First, take nine qubits with an initial state of |0〉 and multiply them together as a tensor product:

(1) $$|C_{0}\rangle ={|0\rangle }_{1}⊗{|0\rangle }_{2}⊗{|0\rangle }_{3}⊗{|0\rangle }_{4}⊗{|0\rangle }_{5}⊗{|0\rangle }_{6}⊗{|0\rangle }_{7}⊗{|0\rangle }_{8}⊗{|0\rangle }_{9}$$

Then, apply the Hadamard gate to the qubits 1, 2, and 3, and the system transforms to $|C_{1}\rangle$:

(2) $$\begin{array}{cc} |C_{1}\rangle & ={\frac{|0\rangle +|1\rangle }{\sqrt{2}}}_{1}⊗{\frac{|0\rangle +|1\rangle }{\sqrt{2}}}_{2}⊗{\frac{|0\rangle +|1\rangle }{\sqrt{2}}}_{3}⊗{|0\rangle }_{4}⊗{|0\rangle }_{5}⊗{|0\rangle }_{6}⊗{|0\rangle }_{7}⊗{|0\rangle }_{8}⊗{|0\rangle }_{9}\\ & =\frac{1}{2\sqrt{2}}(|000000000\rangle ⊗|001000000\rangle ⊗|010000000\rangle ⊗|011000000\rangle \\ & {+⊗|100000000\rangle ⊗|101000000\rangle ⊗|110000000\rangle ⊗|111000000\rangle )}_{123456789} \end{array}$$

Next, we need to apply the CNOT operation. The control bit is Qubit 1, and the target bits are Qubit 5 and Qubit 6. The control bit is Qubit 2, and the target bit is 7. The control bit is Qubit 3, and the target bits are 4, 8, and 9.

(3) $$\begin{array}{cc} |C_{2}\rangle & =\frac{1}{2\sqrt{2}}(|000000000\rangle ⊗|001100011\rangle ⊗|010000100\rangle ⊗|011100111\rangle \\ & {+⊗|100110000\rangle ⊗|101111011\rangle ⊗|110011100\rangle ⊗|111111111\rangle )}_{123456789} \end{array}$$

Now, we have an entangled quantum channel, which is then used for RSP and QT.

### 2.3. Controlled Remote State Preparation and Quantum Teleportation Scheme

Consider using the above nine-qubit entangled states as a quantum channel for multi-party communication. If Alice acts as the sender, Alice wants to remotely prepare any one-qubit and any two-qubit quantum states from Bob and Charlie, respectively, and transmit arbitrary two-qubit quantum states to David via quantum teleportation. Eve acts as an overseer. Alice, Bob, Charlie, David, and Eve share the nine-qubit entangled channels given in Equation (3), where the qubits (1,2,3) of the channels belong to Alice and are marked with bits $a_{0}$, $a_{1}$, and $a_{2}$ respectively, and Eve owns qubits (4), marked with *e*. The qubits (5,6) are in David’s hands, and they are labeled $d_{1}$ and $d_{2}$; the qubits (7) and (8,9) belong to Bob and Charlie, respectively, and are labeled *b* and $c_{1}$ and $c_{2}$, respectively. We can also rewrite the channel given in Equation (3) as follows:

$$\begin{array}{cc} {|C\rangle }_{a_{0}a_{1}a_{2}ed_{1}d_{2}bc_{1}c_{2}} & =\frac{1}{2\sqrt{2}}(|000000000\rangle ⊗|001100011\rangle ⊗|010000100\rangle \\ & ⊗|011100111\rangle +⊗|100110000\rangle ⊗|101111011\rangle \\ & {⊗|110011100\rangle ⊗|111111111\rangle )}_{a_{0}a_{1}a_{2}ed_{1}d_{2}bc_{1}c_{2}} \end{array}$$

Step 1: Suppose Alice has an unknown quantum state of two qubits, which can be written as follows:

$${|\theta_{1}\rangle }_{A_{1}A_{2}}=m|00\rangle +n|11\rangle$$

where *m* and *n* are complex numbers and satisfy ${\left|m\right|}^{2}+{\left|n\right|}^{2}=1$. Alice wants to send the state ${|\theta_{1}\rangle }_{A_{1}A_{2}}$ to David. At the same time, Alice also wants to remotely prepare a single-qubit state and a two-qubit state for Bob and Charlie, respectively, written as:

$$|\theta_{2}\rangle =a|0\rangle +b|1\rangle$$

$$|\theta_{3}\rangle =c|00\rangle +d|11\rangle$$

where *a*, *b* and *c*, *d* are also complex numbers and satisfy ${\left|a\right|}^{2}+{\left|b\right|}^{2}=1$ and ${\left|c\right|}^{2}+{\left|d\right|}^{2}=1$. The quantum state of the entire system can be expressed in the following form:

$$\begin{array}{cc} {|\tau_{0}\rangle }_{A_{1}A_{2}a_{0}a_{1}a_{2}ed_{1}d_{2}bc_{1}c_{2}} & ={|\theta_{1}\rangle }_{A_{1}A_{2}}⊗{|C\rangle }_{a_{0}a_{1}a_{2}ed_{1}d_{2}bc_{1}c_{2}}\\ & =\left(m|00\rangle +n|11\rangle \right)⊗(\frac{1}{2\sqrt{2}}(|000000000\rangle ⊗|001100011\rangle \\ & ⊗|010000100\rangle ⊗|011100111\rangle ⊗|100110000\rangle ⊗|101111011\rangle \\ & ⊗|110011100\rangle ⊗|111111111\rangle )) \end{array}$$

Step 2: Alice performs a three-qubit measurement of the qubits ($A_{1},A_{2},a_{0}$) based on the following basis:

(4) $$\begin{array}{c} |\phi_{1}\rangle =\frac{1}{\sqrt{2}}\left(|000\rangle +|111\rangle \right)\\ |\phi_{2}\rangle =\frac{1}{\sqrt{2}}\left(|000\rangle -|111\rangle \right)\\ |\phi_{3}\rangle =\frac{1}{\sqrt{2}}\left(|001\rangle +|110\rangle \right)\\ |\phi_{4}\rangle =\frac{1}{\sqrt{2}}\left(|001\rangle -|110\rangle \right) \end{array}$$

After taking Equation (4) for the measurement, Alice can obtain one of the four measurements, each with a one-in-four probability. The results of the remaining qubits are shown below. Now, the quantum state will be converted to $|\tau_{1}\rangle$:

$$\begin{array}{cc} |\tau_{1}\rangle & =\frac{1}{4}[{|\phi_{1}\rangle }_{A_{1}A_{2}a_{0}}⊗(m|00000000\rangle +m|01100011\rangle +m|10000100\rangle \\ & +m|11100111\rangle +n|00011000\rangle +n|01111011\rangle +n|10011100\rangle +n|11111111\rangle )\\ & +{|\phi_{2}\rangle }_{A_{1}A_{2}a_{0}}⊗(m|00000000\rangle +m|01100011\rangle +m|10000100\rangle \\ & +m|11100111\rangle -n|00011000\rangle -n|01111011\rangle -n|10011100\rangle -n|11111111\rangle )\\ & +{|\phi_{3}\rangle }_{A_{1}A_{2}a_{0}}⊗(m|00011000\rangle +m|01111011\rangle +m|10011100\rangle +m|11111111\rangle \\ & +n|00000000\rangle +n|01100011\rangle +n|10000100\rangle +n|11100111\rangle )\\ & +{|\phi_{4}\rangle }_{A_{1}A_{2}a_{0}}⊗(m|00011000\rangle +m|01111011\rangle +m|10011100\rangle +m|11111111\rangle \\ & -n|00000000\rangle -n|01100011\rangle -n|10000100\rangle -{n|11100111\rangle )]}_{a_{1}a_{2}ed_{1}d_{2}bc_{1}c_{2}} \end{array}$$

Step 3: Alice performs a two-qubit measurement of the qubits ($a_{1},a_{2}$) on the following basis:

(5) $$\begin{array}{c} |\psi_{1}\rangle =ac|00\rangle +ad|01\rangle +bc|10\rangle +bd|11\rangle \\ |\psi_{2}\rangle =ad|00\rangle -ac|01\rangle +bd|10\rangle -bc|11\rangle \\ |\psi_{3}\rangle =bc|00\rangle +bd|01\rangle -ac|10\rangle -ad|11\rangle \\ |\psi_{4}\rangle =bd|00\rangle -bc|01\rangle -ad|10\rangle +ac|11\rangle \end{array}$$

The measurement basis given by Equation (5) is linearly independent, and after measurement, the remaining unmeasured qubits $ed_{1}d_{2}bc_{1}c_{2}$ will be transformed into $|\tau_{2}\rangle$; the equation for $|\tau_{2}\rangle$ is shown in Equation (A1), Appendix A.

Step 4: Eve makes the von Neumann measurement with the base |+〉,|−〉 on the qubit e. This is the last measurement, and Eve tells David the measurement result through the classical channel; the measurement basis is as follows:

(6) $$\begin{array}{c} |+\rangle =\frac{1}{\sqrt{2}}\left(|0\rangle +|1\rangle \right)\\ |-\rangle =\frac{1}{\sqrt{2}}\left(|0\rangle -|1\rangle \right) \end{array}$$

Now, we can imagine a situation where, if Alice’s measurement is $|\phi_{3}\rangle$ and $|\psi_{4}\rangle$, then after Alice sends the measurement to David, Bob, and Charlie over the classical channel, the remaining unmeasured qubits $ed_{1}d_{2}bc_{1}c_{2}$ will collapse into the following form:

$$\begin{array}{cc} |\tau^{2}\rangle & =\frac{1}{4}|\phi_{3}\rangle ⊗|\psi_{4}\rangle ⊗(mbd|011000\rangle -mbc|111011\rangle -mad|011100\rangle +mac|111111\rangle \\ & {+nbd|000000\rangle -nbc|100011\rangle -nad|000100\rangle +nac|100111\rangle )}_{ed_{1}d_{2}bc_{1}c_{2}} \end{array}$$

Eve only needs to make one von Neumann measurement and, then, tell David the result; if her measurement is |−〉, then the remaining unmeasured qubits $d_{1}d_{2}bc_{1}c_{2}$ will collapse into $|\tau^{3}\rangle$:

$$\begin{array}{cc} |\tau^{3}\rangle & =\frac{1}{4\sqrt{2}}|\phi_{3}\rangle ⊗|\psi_{4}\rangle ⊗|-\rangle ⊗(mbd|11000\rangle +mbc|11011\rangle -mad|11100\rangle -mac|11111\rangle \\ & +nbd|00000\rangle +nbc|00011\rangle -nad|00100\rangle -nac|00111\rangle )\\ & =\frac{1}{4\sqrt{2}}|\phi_{3}\rangle ⊗|\psi_{4}\rangle ⊗|-\rangle ⊗\left(m|11\rangle +n|00\rangle \right)⊗\left(b|0\rangle -a|1\rangle \right)⊗\left(d|00\rangle -c|11\rangle \right) \end{array}$$

Step 5: After Alice and Eve send the measurement results, David does not know the quantum state Alice wants to transmit due to quantum teleportation, so he needs to wait until Alice and Eve send the measurement results to David via classical channels, and then, he needs a specific unitary operation to transform the qubits he holds. While Bob and Charlie are using remote quantum state preparation, they both know what quantum state Alice is preparing, so Bob and Charlie need to use the appropriate unitary operation to achieve Alice’s quantum state preparation at their location. Some unitary operators are shown in Equation (7):

(7) $$\begin{array}{c} X=\left[\begin{array}{cc} 0 & 1\\ 1 & 0 \end{array}\right],Z=\left[\begin{array}{cc} 1 & 0\\ 0 & -1 \end{array}\right],Y=\left[\begin{array}{cc} 0 & -i\\ i & 0 \end{array}\right] \end{array}$$

For the example above, David uses the operator X⊗X to restore the qubits $d_{1}d_{2}$ to the original quantum state sent by Alice. Bob and Charlie use the operators ZX and ZX⊗X for qubits *b* and $c_{1}c_{2}$, respectively, to obtain the initial quantum state prepared by Alice at Bob and Charlie, respectively. The complete multi-party controlled quantum teleportation and remote quantum state preparation schemes are completed at this point. The detailed measurement results of Alice and Eve and the unitary operations required by Bob, Charlie, and David are shown in Table A1.

## 3. Experimental Verification

Currently, some researchers are utilizing the IBM Quantum platform for experimental quantum communication schemes. The IBM Quantum platform, initiated and developed by IBM, is one of the leading platforms in the field of quantum computing. It provides researchers with quantum computers with different numbers of qubits and simulated quantum computers to meet the simulation requirements of quantum communication systems at various scales and complexities. Additionally, the IBM platform provides a wealth of quantum computing programming interfaces and tools, such as Qiskit, which facilitate the development and experimentation of quantum communication simulations. Through these tools, users can design and implement quantum communication protocols, simulate quantum channel transmissions, and test quantum-error-correction schemes.

The proposed scheme employs a combination of two protocols: quantum teleportation and remote quantum state preparation. It utilizes various quantum gate circuits, and with the aid of the IBM Quantum platform, it can effectively achieve the channel preparation, remote quantum state preparation, and quantum state transmission required for this communication scheme.

### 3.1. Experimental Verification of Controlled Quantum Communication Protocols Based on Multiple Parties

Our experiments were carried out on the IBM Quantum platform, and IBM’s ibmq-qasm-simulator was used for the simulation experiments. The experiment is divided into two parts: the experimental channel preparation and the experimental communication protocol implementation. The experimental environment is shown in Table A2. We implemented the channel preparation of the above theory and the whole process of the protocol through experiments. The simulation experiment was designed to be carried out in an ideal state without quantum computing attacks. The actual IoT multi-device communication and communication security analysis are described in detail in Section 4 and Section 5.

### 3.2. Experiments on Channel Preparation

We need to prepare the channel circuit:

$$\begin{array}{cc} {|C\rangle }_{a_{0}a_{1}a_{2}ed_{1}d_{2}bc_{1}c_{2}} & =\frac{1}{2\sqrt{2}}(|000000000\rangle ⊗|001100011\rangle ⊗|010000100\rangle ⊗|011100111\rangle \\ & {+⊗|100110000\rangle ⊗|101111011\rangle ⊗|110011100\rangle ⊗|111111111\rangle )}_{a_{0}a_{1}a_{2}ed_{1}d_{2}bc_{1}c_{2}} \end{array}$$

$q_{2}$ to $q_{1}0$ represent the qubits ($a_{0}$, $a_{1}$, $a_{2}$, *e*, $d_{1}$, $d_{2}$, *b*, $c_{1}$, $c_{2}$), and the initial state from $q_{2}$ to $q_{10}$ is |0〉.

**Figure 1:**
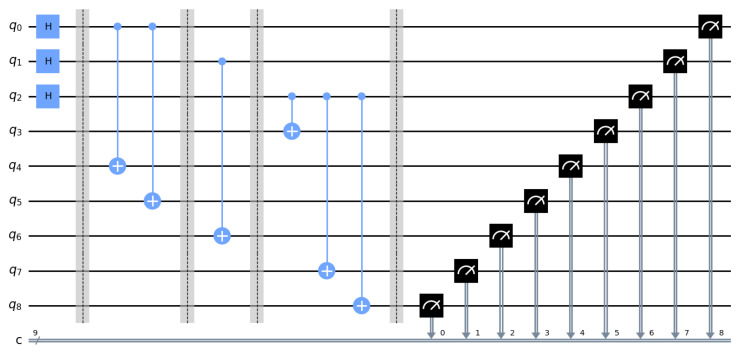
This circuit builds a nine-qubit entanglement channel. From left to right, $q_{2}$ to $q_{1}0$ represent the qubits ($a_{0}$, $a_{1}$, $a_{2}$, *e*, $d_{1}$, $d_{2}$, *b*, $c_{1}$, $c_{2}$), and the initial state from $q_{2}$ to $q_{10}$ is |0〉. The first part is the H gate, the middle parts 2, 3, and 4 are the CNOT operation, and the fifth part is the measurement operation. The number pointed by the arrow of the measurement mark represents the classical bit number. The diagram shows the circuit diagram process of the channel preparation experiment.

**Figure 2:**
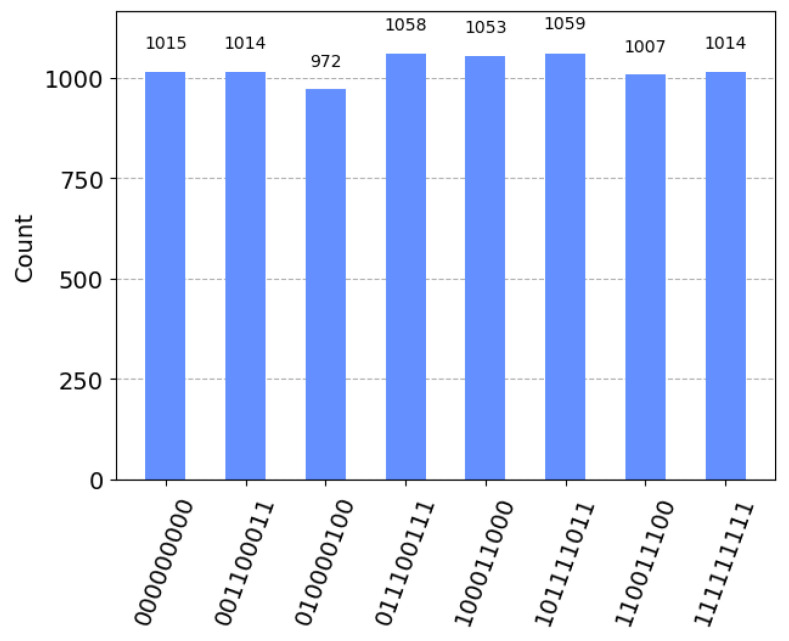
After three H gates and six CNOT operations, the channel system is entangled; the horizontal coordinate represents the corresponding quantum state that may collapse, and the vertical coordinate represents the number of collapses of each quantum state. The figure shows that the collapse probability of each quantum state is basically the same, which reflects the rationality of the entangled channel.

First, $q_{2},q_{3}$ and $q_{4}$ are prepared using the H gate, and then, six CNOT operations are used to complete the channel preparation. Figure 1 shows the entire process of channel preparation. Figure 2 shows the probability obtained by measuring each quantum state. A total of 8192 shots were made, and the probability of collapsing into eight possible quantum states was basically equal. The circuit and measurement experiment verified the entanglement degree and reliability of the channel.

### 3.3. Experimental Quantum Teleportation and Remote Quantum State Preparation Process

A total of 11 particles are required for the entire transmission process. $q_{0}$ and $q_{1}$ belong to Alice, while $q_{2}$ through $q_{10}$ are channel particles. Eleven classical registers are used during the circuit to store the measured values.

The transmission process is divided into three parts, including the initialization of the quantum state, the measurement operation performed separately, and the corresponding unitary operation at the end.

The first is the initialization phase. Since we want to transmit arbitrary quantum states, we must build a quantum gate that initializes the coefficients. For Alice, her transmission to Bob, Charlie, and David requires quantum state initialization, respectively, in order to achieve any quantum state transmission process. Here, we used Qiskit’s Operator function to do this. Assuming the coefficients from Alice to David are $m=\sqrt{\frac{6}{7}}$ and $n=\frac{1}{\sqrt{7}}$, then the U gate can be expressed as:

$$\frac{1}{\sqrt{7}}\left[\begin{array}{cc} \sqrt{6} & 1\\ 1 & -\sqrt{6} \end{array}\right]$$

Similarly, suppose we need to initialize the quantum states to be passed to Bob and Charlie, whose coefficients are:

$$\frac{1}{\sqrt{2}}\left[\begin{array}{cc} 1 & 1\\ 1 & -1 \end{array}\right],\frac{1}{\sqrt{11}}\left[\begin{array}{cc} \sqrt{10} & 1\\ 1 & -\sqrt{10} \end{array}\right]$$

After initializing each coefficient, we can take the measurement step; Alice takes two measurements, namely the three-qubit measurement and the two-qubit measurement, while Eve takes only one von Neumann measurement; in the three-qubit measurement, we measure $A_{1}$, $A_{2}$ and $a_{0}$, corresponding to $q_{0}$, $q_{1}$, and $q_{2}$, and in the two-qubit measurement, we measure $a_{1}$ and $a_{2}$, corresponding to the circuit qubits $q_{3}$ and $q_{4}$. Eve performs a von Neumann measurement of the particle *e* corresponding to $q_{5}$.

**Figure 3:**
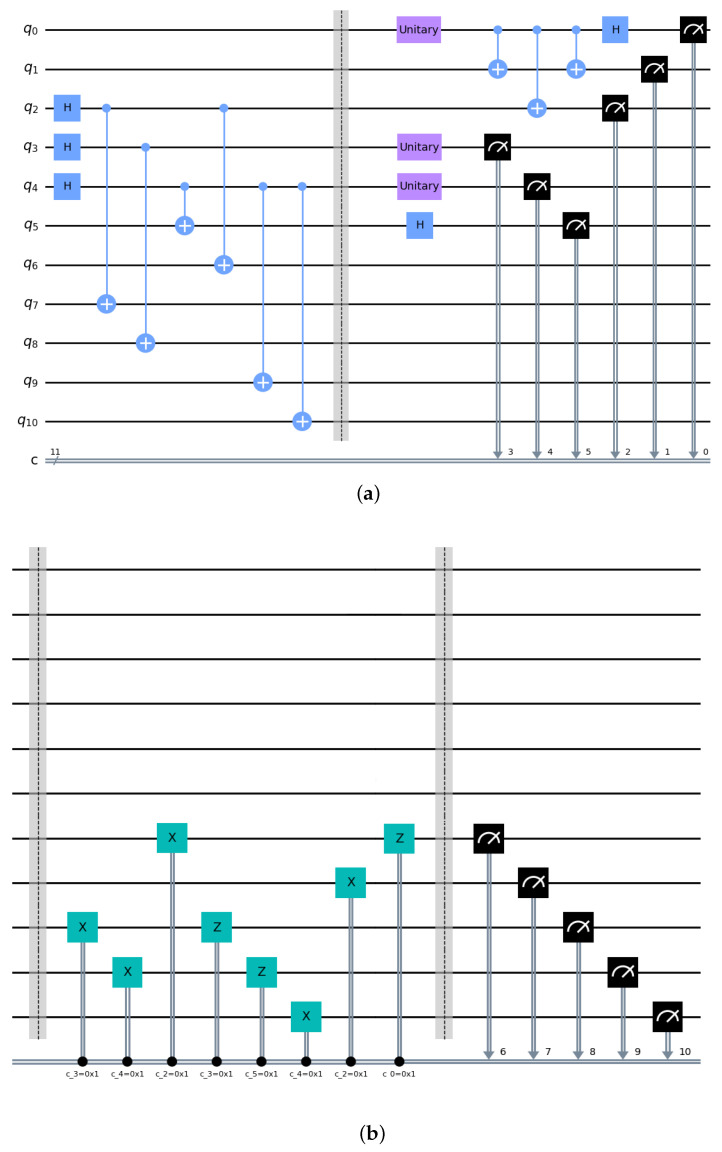
This is a circuit diagram of controlled remote state preparation and quantum teleportation using a nine-qubit entangled channel. From $q_{0}$ to $q_{10}$ represent the qubits ($A_{1}$, $A_{2}$, $a_{1}$, $a_{2}$, *e*, $d_{1}$, $d_{2}$, *b*, $c_{1}$, $c_{2}$). Marked by the dividing line, the first part of Figure (**a**) is the channel preparation; the second part is the preparation and initialization of the quantum state to be transmitted; the first part of Figure (**b**) is the corresponding unitary operation; the second part is the measurement process. “H” indicates the H gate; the light blue symbol “+” indicates the target bit; the light blue dot represents the control bit; both the Init gate and Unitary gate are initialization operations for quantum states to enable any quantum state transmission. The black mark represents the measurement operation; the X and Z identifiers represent X operations and Z operations, whose matrix form is indicated in Equation (7). The two diagrams show the complete protocol process.

Bob, Charlie, and David then need to perform the appropriate unitary operations on their particles *b*, $c_{1}$, $c_{2}$, $d_{1}$, and $d_{2}$ to achieve the final process of remote state preparation and quantum teleportation. The whole circuit diagram is shown in Figure 3, including the channel preparation, the preparation and initialization of the quantum state to be transmitted, and the circuit for the final unitary operation.

**Figure 4:**
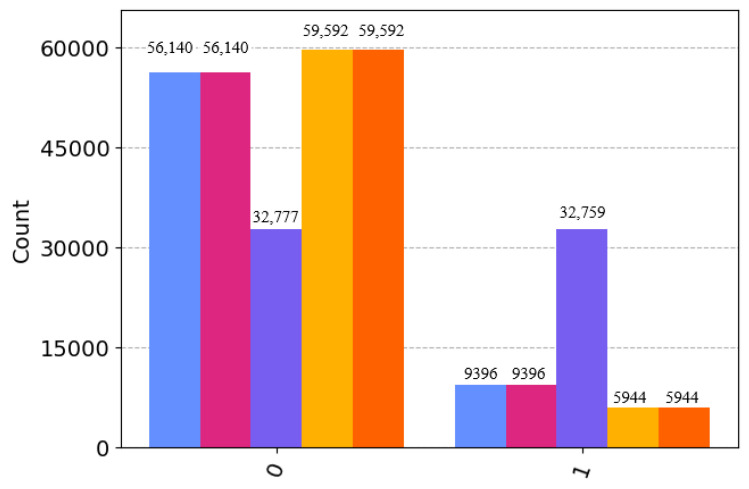
The 0 and 1 represent |0〉 and |1〉. The first two bars of 0 and 1, respectively, are David’s experimental results; the middle bar is Bob’s experimental results; the last two bars are Charlie’s experimental results, which are very close to the expected results.

For the coefficients mentioned above, the quantum state $\sqrt{\frac{6}{7}}|00\rangle +\frac{1}{\sqrt{7}}|11\rangle$ should be six-times more likely to measure |00〉 than |11〉. Similarly, for the quantum states $\frac{1}{\sqrt{2}}|0\rangle +\frac{1}{\sqrt{2}}|1\rangle$ and $\sqrt{\frac{10}{11}}|00\rangle +\frac{1}{\sqrt{11}}|11\rangle$, the former is equally likely to measure |0〉 and |1〉, while the latter is ten-times more likely to measure |00〉 than |11〉. We tested the above process in the IBM Quantum platform qasm-simulator. To make the experimental results more accurate and reduce the statistical error, the scheme’s performance was evaluated comprehensively. In this article, parameter “shots” was set to 65,536; too large a value may cause performance problems. The specific measurement results of the experiment are shown in Figure 4.

The experiment showed that the measured results of Bob, Charlie, and David after restoring the quantum state through their respective unitary operations were basically consistent with the theoretically calculated probability values, and the entire experimental process of the transmission protocol and the preparation protocol is complete.

## 4. Application Analysis of Quantum Secure Communication Based on Multi-Devices IoT

The preparation based on controlled quantum teleportation and remote quantum states has many application scenarios:

Quantum secure communication: This can be used to enable secure communication in the IoT. Through the transmission and preparation of quantum states, the secure transmission of encryption keys can be achieved, thus ensuring the confidentiality and integrity of communication. This has important implications regarding sensitive data transfer and privacy protection, such as financial transactions, medical records, and personal authentication.

Quantum sensing networks: IoT sensor networks can use QT and RSP to enable efficient sensor data transmission and processing. By transmitting quantum states to remote nodes, distributed perception and collaborative processing can be achieved, thereby improving the performance and capability of sensor networks. This has potential applications in areas such as environmental monitoring, intelligent transportation, and agriculture.

Distributed quantum computing: QT and RSP can enable distributed quantum computing in the IoT. By preparing quantum states remotely, different devices can work together on quantum computing tasks, thus enabling the acceleration and expansion of distributed computing. This has potential applications in optimization problem-solving, machine learning, and large-scale data processing.

Quantum authentication and authentication in the IoT: Quantum teleportation and remote quantum state preparation can be applied to IoT’s authentication and authentication process. By taking advantage of the uniqueness and non-repudiation of quantum states, a more-secure and -reliable authentication mechanism can be achieved, thereby preventing deception and forgery.

**Figure 5:**
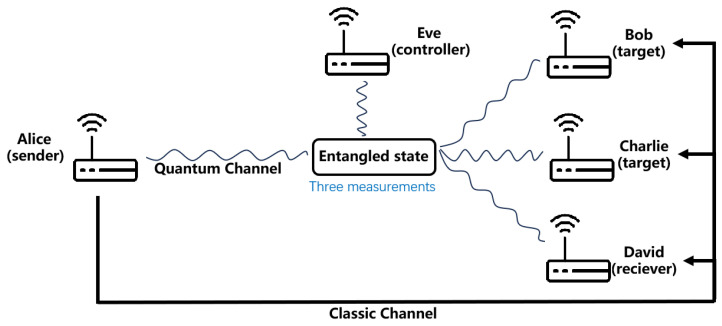
Alice represents the sending device; David represents the receiving device; Bob and Charlie represent the target device for the preparation of the quantum state; Eve is the control device. The figure shows the use of quantum channels, which are used to transmit and measure quantum states, and classical channels, which are used to transmit unitary operations.

This part mainly introduces the secure communication process of quantum teleportation and remote quantum state preparation in the IoT. In the actual multi-party communication of the IoT, the five-qubit QT and RSP based on the nine-qubit quantum channel have a broad application prospect. This part introduces the application scenario analysis of the five-qubit QT based on the nine-qubit quantum channel transmission and the RSP in the actual multi-party communication of the IoT and uses QT to achieve secure quantum information transmission in the multiple devices communication of the IoT. Suppose there are five devices: Devices A (Alice), B (Bob), C (Charlie), D (David), and E (Eve), and Alice wants to transmit the two-qubit state to David via QT and prepare one qubit and two qubits for Bob and Charlie, respectively. As the controller, Eve monitors and controls the transmission, aiming to ensure the security of the transmission. Figure 5 shows the use of quantum channels, which are used to transmit and measure quantum states, and classical channels, which are used to transmit unitary operations.

Step 1: Prepare the devices:

In practical IoT multi-party communication for QT and remote quantum state preparation, each device must be equipped with a quantum computer to generate, manipulate, and measure qubits. These devices can be nodes or end devices in the IoT, such as sensors, smart devices, or communication terminals.

Step 2: Qubit channel preparation:

In order to realize QT and remote quantum state preparation, a quantum channel must be prepared first. The initial quantum states can be entangled together to establish entangled quantum channels by applying appropriate quantum gate operations. This entangled quantum channel will be used to transmit information about quantum states.

Step 3: Initialize the quantum state of the transmission:

As the sender, Alice uses her quantum computer to prepare the two-qubit state to transmit to David. This quantum state can be initialized using appropriate unitary operations such as U gates.

Step 4: Measurement base selection and measurement:

In QT and RSP, Alice needs to choose the measurement basis given in Formula (5) to measure the tensor product of the quantum state and quantum channel that she has. However, this measurement process does not immediately provide classical bit information. To obtain classical bit information, the quantum states of Bob and Charlie also need to be two-qubit-measured against the tensor product of the channel quantum state, while Eve needs to be single-qubit-measured against the corresponding qubit in the channel.

Step 5: Quantum state transmission and preparation:

Alice sends David the quantum state information with the corresponding unitary operation through the classical communication channel. David can use his quantum computer to perform the corresponding operation to recover the quantum state information sent by Alice. At the same time, Bob and Charlie can also use their quantum computer to perform unitary operations on the quantum states they measure in the channel to achieve the quantum states Alice prepared at their place.

Step 6: Security assurance:

As a controller, Eve can monitor and control communication links. If the communication link is not secure, Eve can interrupt the communication link to ensure the security of the transmission. In the subsequent communication process, Eve does not perform the corresponding single-qubit measurement, thus protecting the security of quantum information.

By building entangled quantum channels and using appropriate measurement bases, Alice can send states to Bob, Charlie, and David and prevent eavesdropping by adding a control device, Eve. QT and RSP enable secure information transmission in the IoT multi-device communication. This method can be applied to multi-party communication scenarios in the IoT to ensure the confidentiality and integrity of quantum information in communication, while also helping to protect sensitive information and improve the security of the IoT system. However, for larger-scale and complex IoT systems, further research and development of quantum security protocols and technologies are needed to deal with potential attacks and security threats.

## 5. Security Analysis

In this section, we explore the security of this solution in the IoT. Suppose there is an eavesdropper except for Device A (Alice), Device B (Bob), Device C (Charlie), Device D (David), and controller Device E (Eve). In that case, the eavesdropper has several attack methods, namely intercept–replace–resend attack and intercept–measure–resend attack; we verified the security of this scheme by studying these two attack methods. Since this scheme has two methods, namely remote quantum state preparation and QT, we discuss the security of Alice’s quantum state preparation at Bob and Charlie and the security of Alice’s quantum state sending to David through QT.

Since there is a substitution attack or measurement attack during the remote quantum state preparation, the receiver to perform remote preparation will know the prepared quantum state in advance, and the prepared quantum state does not match the target quantum state, indicating that the protocol has an attack, so the attack will be detected. We need to add a way to improve security for the transmission process that Alice sends to David. After Alice sends her measurement to David over the classical channel, in the process, we can add a sequence of quantum states ${|01\rangle }_{ij},{|10\rangle }_{ij}$ to combine it with the measurement Alice sends to David and perform the appropriate CNOT operation. If she wants to send her measurement results to David, she needs to select any state in a sequence and insert it at any point in transmitting the quantum state, and then, Alice sends the quantum state to David through the classical channel. David only needs to use the quantum state sent by Alice and the measurement results to know the quantum state sent by Alice and whether the QT protocol is eavesdropped.

### 5.1. Intercept–Replace–Resend Attack

Suppose there is an intercept–replace–resend attack between Alice, Bob, and Charlie since Alice uses remote quantum state preparation. In that case, Bob and Charlie know in advance the quantum state Alice will prepare in their place, so once the replace–resend occurs, Bob and Charlie cannot restore the remaining unmeasured qubits to the quantum state Alice wants to prepare by using the corresponding unitary operator, so it can be found that the enemy attacks the communication. Still, in this process, if the classical channel has been eavesdropped, information will be leaked. The enemy will directly use the information.

Alice sends her measurements to David, assuming that the eavesdropper is trying to intercept Alice’s quantum state and the quantum state Alice intended to transmit to David in the first place. However, Alice has changed the conversion rule of this quantum state, so when David receives the false quantum state replaced by the eavesdropper, he first checks the insertion position and conversion rule told by Alice in advance and then performs the corresponding CNOT operation to obtain the converted quantum state. The quantum state sent by Alice over the classical channel is then compared with the quantum state obtained by David, who also realizes that the information he received is wrong, and the eavesdropper’s attack fails.

### 5.2. Intercept–Measure–Resend Attack

Similarly, since Bob and Charlie know in advance the quantum state to be prepared by Alice and the quantum state is informed by Alice through the classical channel, Bob and Charlie will find it invalid if an attack occurs during the remote quantum state preparation process. When Alice sends information to David via QT, suppose there is an eavesdropper who tries to intercept the quantum state Alice sends to David when Alice sends the measurement result to the receiver, David, and performs an appropriate measurement on that quantum state, then the information Alice is trying to obtain is sent to David. However, the eavesdropper’s interception will not succeed because Alice has already performed a proper transformation of the quantum state before sending the measurement results. Since the eavesdropper does not know Alice’s conversion rules, no matter how it performs the measurement, it will not obtain any information about Alice’s transmission.

For example, if the measurements of Alice are $|\phi_{1}\rangle$ and $|\psi_{1}\rangle$ and the measurement of Eve is |+〉, then David’s result is ${\left(m|00\rangle +n|11\rangle \right)}_{d_{1}d_{2}}$. If Alice chooses the sequence ${|01\rangle }_{ij}$, then $d_{1},d_{2}$ are the control bits, and *i* and *j* are the target bits, then the quantum state can be rewritten as:

$$\left(m|00\rangle +n|11\rangle \right)|01\rangle \left(a|0\rangle +b|1\rangle \right){\left(c|00\rangle +d|11\rangle \right)}_{d_{1}d_{2}ijbc_{1}c_{2}}$$

If the CNOT operation is performed, $d_{1}$ controls *i*, and $d_{2}$ controls *j*, then the quantum state can be rewritten as:

$$\left(m|0001\rangle +n|1110\rangle \right)\left(a|0\rangle +b|1\rangle \right){\left(c|00\rangle +d|11\rangle \right)}_{d_{1}d_{2}ijbc_{1}c_{2}}$$

Then, Alice transmits this quantum state to David through the classical channel, where $d_{1}d_{2}ij$ is David’s, and the conversion rule is known only to Alice and David. Assuming that the eavesdropper performs a single particle measurement on it, the entangled quantum state sequence will be affected. If the measurement attack occurs, the quantum state sequence will be affected. David would have detected it.

As another example, if Alice’s measurement result is $|\phi_{2}\rangle$ and $|\psi_{3}\rangle$ and Eve’s measurement result is |−〉, through the previous analysis, We can obtain David’s result as ${\left(m|00\rangle -n|11\rangle \right)}_{d_{1}d_{2}}$, and if Alice chooses ${|11\rangle }_{ij}$, we also take $d_{1},d_{2}$ as the control bits and *i* and *j* as the target bits. The quantum state can be rewritten as:

$$\left(m|00\rangle -n|11\rangle \right)|11\rangle ⊗\left(b|0\rangle -a|1\rangle \right)⊗{\left(c|00\rangle -d|11\rangle \right)}_{d_{1}d_{2}ijc_{1}c_{2}}$$

The same as above, we perform the CNOT operation, and the quantum state changes:

$$\left(m|0011\rangle -n|1100\rangle \right)⊗\left(b|0\rangle -a|1\rangle \right)⊗{\left(c|00\rangle -d|11\rangle \right)}_{d_{1}d_{2}ijc_{1}c_{2}}$$

Similarly, if the eavesdropper performs a single-quantum measurement or double-qubit measurement on the quantum state sent by Alice and the sequence of quantum states sent together with Alice, the original transmission form will be changed, leading to the discovery of the attack.

The results of the above two tests showed that neither an intercept–measure–resend attack nor an intercept–replace–resend attack can obtain valid information while transmitting quantum states. Therefore, our method is proven to be safe and reliable. By taking additional steps to ensure the security of the transmitted quantum state, we can further improve the security of QT and enable it to be used for a wide range of secure communication applications. Moreover, the security of QT is not limited to detecting and preventing eavesdropping attacks. It can also be used to distribute keys between two parties and, then, for secure communication. In this case, Alice and Bob would use QT to transmit the shared key, which would then be used to encrypt and decrypt the messages sent between them. Since any attempt to intercept the key will be detected, the technology provides a high degree of security for sensitive communications of IoT multiple devices, greatly improving the ability to prevent quantum computing attacks.

## 6. Protocol Comparison and Analysis

In this section, we compare and analyze related protocols from the aspects of quantum channel selection, whether there is a supervisor, and resource utilization. The formula for transmission efficiency was constructed based on the number of particles transmitted, the number of channel particles, the number of classical bit particles used, and the number of auxiliary particles required:

$$\eta =\frac{c}{p+q+t}$$

*c* is the number of particles transmitted or prepared; *p* is the number of particles required by the channel; *q* is the number of classical bits used in the transmission process; *t* is the number of auxiliary particles used. Five particles are transmitted in our scheme, while the channel uses nine particles. In the whole transmission process, classic bits use six due to the need to use classical bits to perform the corresponding unitary operation of the receiver. Our efficiency is 33.3%. The formula for transmission efficiency mentioned in this article and other comparative studies do not consider information transmission outside of the protocol. Therefore, this formula applies only within the specific transmission protocol context.

Table 1 shows the schemes for comparison in this paper.

The protocol used in [38] adopted an eight-qubit channel to realize bidirectional transmission of any two-qubit state, but the transmission efficiency formula given in [38] is inconsistent with the transmission efficiency calculation formula given by us. Here, according to the transmission efficiency calculation formula adopted in this paper, $\eta =\frac{4}{8+8+0}=25\%$, the efficiency of [38] should be 25%. In [39], they used a five-qubit channel to transmit three-qubit quantum states in a bidirectional controlled manner, with an efficiency of 30%. In [24], consistent with our scheme, a nine-qubit channel was also adopted for communication, and a total of five-qubit quantum states were also transmitted bi-directionally. The paper [19] used an 8-qubit channel to transmit a 3-qubit channel, with a transmission efficiency of 3/16, which is relatively low. The transmission form of the paper [18] was the same as that of the paper [39], but the transmission efficiency was not improved. However, we reduced the amount of classical bits, and it is a controlled quantum teleportation with relatively higher efficiency.

Finally, different from the scheme adopted in the above paper, our scheme aimed to realize a multi-receiver multi-party communication, which is also one of the differences from the above schemes. Through the comparison and analysis, the advantages of this scheme were higher efficiency and multi-party communication.

## 7. Conclusions

This paper proposed a multi-party-based scheme for controlled remote quantum state preparation and quantum teleportation, which realizes multi-device communication in the IoT using a nine-qubit entanglement channel. The scheme was explained and verified in detail from the two aspects of theory and experiment, and the safety and efficiency of the scheme were analyzed. This paper’s main contributions and discoveries were as follows: a novel nine-qubit entangled channel was constructed, which can simultaneously achieve controlled remote quantum state preparation and quantum teleportation, expanding the possibility and diversity of quantum communication. The channel preparation, measurement operation, and unitary operation of the scheme were successfully simulated on the IBM Quantum platform, and 8192 shots verified the entanglement degree and reliability of the channel. This paper not only analyzed the steps and methods of the scheme in the multi-device communication of the IoT, but also deduced the effect of the attack on the communication scheme by simulating two different attack modes and analyzed that the scheme has high security and anti-interference ability in the multi-device communication of the IoT. Compared with other schemes, this paper briefly analyzed two aspects of principle and transmission efficiency and pointed out the advantages of this scheme. This scheme can provide a reference for future secure communication of IoT devices and multi-party quantum transmission protocols. Although QT and RSP have advantages in terms of security, they still face other implementation challenges, such as the reliability of the entanglement distribution and the impact of noise and errors. This scheme only considered the ideal noise-free quantum communication scheme. An improvement of this paper may be to discuss the influence of noise and take corresponding measures to deal with the influence of noise.

## Figures and Tables

**Table 1:** Comparison of transmission efficiency results, including the transmission channel, the number of particles transmitted, and whether a controller was added.

Protocol	Teleported Particles	Quantum Channel	Controller (Yes/No)	Efficiency
[38]	Four-qubit	Eight-qubit	No	25%
[39]	Three-qubit	Five-qubit	No	30%
[24]	Five-qubit	Nine-qubit	No	23.8%
[19]	Three-qubit	Eight-qubit	No	18.75%
[18]	Three-qubit	Five-qubit	No	30%
proposed	Five-qubit	Nine-qubit	Yes	33.3%

## Data Availability

The data are contained within the article.

## Abbreviations

The following abbreviations are used in this manuscript:

IoT	Internet of Things
QT	Quantum Teleportation
QCT	Quantum Controlled Teleportation
QKD	Quantum Key Distribution
QSC	Quantum Superdense Coding
QIS	Quantum Information Splitting
ABQT	Asymmetric Bidirectional Quantum Teleportation
RSP	Remote State Preparation

## Appendix A

(A1)
$$\begin{array}{c} |\tau_{2}\rangle =\frac{1}{4}[|\phi_{1}\rangle ⊗|\psi_{1}\rangle ⊗(mac|000000\rangle +mad|100011\rangle +mbc|000100\rangle +mbd|100111\rangle \\ +nac|011000\rangle +nad|111011\rangle +nbc|011100\rangle +nbd|111111\rangle )\\ +|\phi_{2}\rangle ⊗|\psi_{1}\rangle ⊗(mac|000000\rangle +mad|100011\rangle +mbc|000100\rangle +mbd|100111\rangle \\ -nac|011000\rangle -nad|111011\rangle -nbc|011100\rangle -nbd|111111\rangle )\\ +|\phi_{3}\rangle ⊗|\psi_{1}\rangle ⊗(mac|011000\rangle +mad|111011\rangle +mbc|011100\rangle +mbd|111111\rangle \\ +nac|000000\rangle +nad|100011\rangle +nbc|000100\rangle +nbd|100111\rangle )\\ +|\phi_{4}\rangle ⊗|\psi_{1}\rangle ⊗(mac|011000\rangle +mad|111011\rangle +mbc|011100\rangle +mbd|111111\rangle \\ -nac|000000\rangle -nad|100011\rangle -nbc|000100\rangle -nbd|100111\rangle )\\ +|\phi_{1}\rangle ⊗|\psi_{2}\rangle ⊗(mad|000000\rangle -mac|100011\rangle +mbd|000100\rangle -mbc|100111\rangle \\ +nad|011000\rangle -nac|111011\rangle +nbd|011100\rangle -nbc|111111\rangle )\\ +|\phi_{2}\rangle ⊗|\psi_{2}\rangle ⊗(mad|000000\rangle -mac|100011\rangle +mbd|000100\rangle -mbc|100111\rangle \\ -nad|011000\rangle +nac|111011\rangle -nbd|011100\rangle +nbc|111111\rangle )\\ +|\phi_{3}\rangle ⊗|\psi_{2}\rangle ⊗(mad|011000\rangle -mac|111011\rangle +mbd|011100\rangle -mbc|111111\rangle \\ +nad|000000\rangle -nac|100011\rangle +nbd|000100\rangle -nbc|100111\rangle )\\ +|\phi_{4}\rangle ⊗|\psi_{2}\rangle ⊗(mad|011000\rangle -mac|111011\rangle +mbd|011100\rangle -mbc|111111\rangle \\ -nad|000000\rangle +nac|100011\rangle -nbd|000100\rangle +nbc|100111\rangle )\\ +|\phi_{1}\rangle ⊗|\psi_{3}\rangle ⊗(mbc|000000\rangle +mbd|100011\rangle -mac|000100\rangle -mad|100111\rangle \\ +nbc|011000\rangle +nbd|111011\rangle -nac|011100\rangle -nad|111111\rangle )\\ +|\phi_{2}\rangle ⊗|\psi_{3}\rangle ⊗(mbc|000000\rangle +mbd|100011\rangle -mac|000100\rangle -mad|100111\rangle \\ -nbc|011000\rangle -nbd|111011\rangle +nac|011100\rangle +nad|111111\rangle )\\ +|\phi_{3}\rangle ⊗|\psi_{3}\rangle ⊗(mbc|011000\rangle +mbd|111011\rangle -mac|011100\rangle -mad|111111\rangle \\ +nbc|000000\rangle +nbd|100011\rangle -nac|000100\rangle -nad|100111\rangle )\\ +|\phi_{4}\rangle ⊗|\psi_{3}\rangle ⊗(mbc|011000\rangle +mbd|111011\rangle -mac|011100\rangle -mad|111111\rangle \\ -nbc|000000\rangle -nbd|100011\rangle +nac|000100\rangle +nad|100111\rangle )\\ +|\phi_{1}\rangle ⊗|\psi_{4}\rangle ⊗(mbd|000000\rangle -mbc|100011\rangle -mad|000100\rangle +mac|100111\rangle \\ +nbd|011000\rangle -nbc|111011\rangle -nad|011100\rangle +nac|111111\rangle )\\ +|\phi_{2}\rangle ⊗|\psi_{4}\rangle ⊗(mbd|000000\rangle -mbc|100011\rangle -mad|000100\rangle +mac|100111\rangle \\ -nbd|011000\rangle +nbc|111011\rangle +nad|011100\rangle -nac|111111\rangle )\\ +|\phi_{3}\rangle ⊗|\psi_{4}\rangle ⊗(mbd|011000\rangle -mbc|111011\rangle -mad|011100\rangle +mac|111111\rangle \\ +nbd|000000\rangle -nbc|100011\rangle -nad|000100\rangle +nac|100111\rangle )\\ +|\phi_{4}\rangle ⊗|\psi_{4}\rangle ⊗(mbd|011000\rangle -mbc|111011\rangle -mad|011100\rangle +mac|111111\rangle \\ -nbd|000000\rangle +nbc|100011\rangle +nad|000100\rangle -{nac|100111\rangle )]}_{ed_{1}d_{2}bc_{1}c_{2}} \end{array}$$

## Appendix B

**Table A1:** The measurement results of Alice and Eve, the state of the remaining qubits, and the specific unitary operator, where ZX indicates that the Z operation is performed before the X operation is performed.

Alice’s R	Eve’s R	Remaining Unmeasured Qubits	David’s Gate	Bob’s Gate	Charlie’s Gate
$|\phi_{1}\rangle ⊗|\psi_{1}\rangle$	|+〉	(m|00〉+n|11〉)⊗(a|0〉+b|1〉)⊗(c|00〉+d|11〉)	I⊗I	*I*	I⊗I
	|−〉	(m|00〉+n|11〉)⊗(a|0〉+b|1〉)⊗(c|00〉−d|11〉)	I⊗I	*I*	Z⊗I
$|\phi_{2}\rangle ⊗|\psi_{1}\rangle$	|+〉	(m|00〉−n|11〉)⊗(a|0〉+b|1〉)⊗(c|00〉+d|11〉)	Z⊗I	*I*	I⊗I
	|−〉	(m|00〉−n|11〉)⊗(a|0〉+b|1〉)⊗(c|00〉−d|11〉)	Z⊗I	*I*	Z⊗I
$|\phi_{3}\rangle ⊗|\psi_{1}\rangle$	|+〉	(m|11〉+n|00〉)⊗(a|0〉+b|1〉)⊗(c|00〉+d|11〉)	X⊗X	*I*	I⊗I
	|−〉	(m|11〉+n|00〉)⊗(a|0〉+b|1〉)⊗(c|00〉−d|11〉)	X⊗X	*I*	Z⊗I
$|\phi_{4}\rangle ⊗|\psi_{1}\rangle$	|+〉	(m|11〉−n|00〉)⊗(a|0〉+b|1〉)⊗(c|00〉+d|11〉)	ZX⊗X	*I*	I⊗I
	|−〉	(m|11〉−n|00〉)⊗(a|0〉+b|1〉)⊗(c|00〉−d|11〉)	ZX⊗X	*I*	Z⊗I
$|\phi_{1}\rangle ⊗|\psi_{2}\rangle$	|+〉	(m|00〉+n|11〉)⊗(a|0〉+b|1〉)⊗(d|00〉−c|11〉)	I⊗I	*I*	ZX⊗X
	|−〉	(m|00〉+n|11〉)⊗(a|0〉+b|1〉)⊗(d|00〉+c|11〉)	I⊗I	*I*	X⊗X
$|\phi_{2}\rangle ⊗|\psi_{2}\rangle$	|+〉	(m|00〉−n|11〉)⊗(a|0〉+b|1〉)⊗(d|00〉−c|11〉)	Z⊗I	*I*	ZX⊗X
	|−〉	(m|00〉−n|11〉)⊗(a|0〉+b|1〉)⊗(d|00〉+c|11〉)	Z⊗I	*I*	X⊗X
$|\phi_{3}\rangle ⊗|\psi_{2}\rangle$	|+〉	(m|11〉+n|00〉)⊗(a|0〉+b|1〉)⊗(d|00〉−c|11〉)	X⊗X	*I*	ZX⊗X
	|−〉	(m|11〉+n|00〉)⊗(a|0〉+b|1〉)⊗(d|00〉+c|11〉)	X⊗X	*I*	X⊗X
$|\phi_{4}\rangle ⊗|\psi_{2}\rangle$	|+〉	(m|11〉−n|00〉)⊗(a|0〉+b|1〉)⊗(d|00〉−c|11〉)	ZX⊗X	*I*	ZX⊗X
	|−〉	(m|11〉−n|00〉)⊗(a|0〉+b|1〉)⊗(d|00〉+c|11〉)	ZX⊗X	*I*	X⊗X
$|\phi_{1}\rangle ⊗|\psi_{3}\rangle$	|+〉	(m|00〉+n|11〉)⊗(b|0〉−a|1〉)⊗(c|00〉+d|11〉)	I⊗I	ZX	I⊗I
	|−〉	(m|00〉+n|11〉)⊗(b|0〉−a|1〉)⊗(c|00〉−d|11〉)	I⊗I	ZX	Z⊗I
$|\phi_{2}\rangle ⊗|\psi_{3}\rangle$	|+〉	(m|00〉−n|11〉)⊗(b|0〉−a|1〉)⊗(c|00〉+d|11〉)	Z⊗I	ZX	I⊗I
	|−〉	(m|00〉−n|11〉)⊗(b|0〉−a|1〉)⊗(c|00〉−d|11〉)	Z⊗I	ZX	Z⊗I
$|\phi_{3}\rangle ⊗|\psi_{3}\rangle$	|+〉	(m|11〉+n|00〉)⊗(b|0〉−a|1〉)⊗(c|00〉+d|11〉)	X⊗X	ZX	I⊗I
	|−〉	(m|11〉+n|00〉)⊗(b|0〉−a|1〉)⊗(c|00〉−d|11〉)	X⊗X	ZX	Z⊗I
$|\phi_{4}\rangle ⊗|\psi_{3}\rangle$	|+〉	(m|11〉−n|00〉)⊗(b|0〉−a|1〉)⊗(c|00〉+d|11〉)	ZX⊗X	ZX	I⊗I
	|−〉	(m|11〉−n|00〉)⊗(b|0〉−a|1〉)⊗(c|00〉−d|11〉)	ZX⊗X	ZX	Z⊗I
$|\phi_{1}\rangle ⊗|\psi_{4}\rangle$	|+〉	(m|00〉+n|11〉)⊗(b|0〉−a|1〉)⊗(d|00〉−c|11〉)	I⊗I	ZX	ZX⊗X
	|−〉	(m|00〉+n|11〉)⊗(b|0〉−a|1〉)⊗(d|00〉+c|11〉)	I⊗I	ZX	X⊗X
$|\phi_{2}\rangle ⊗|\psi_{4}\rangle$	|+〉	(m|00〉−n|11〉)⊗(b|0〉−a|1〉)⊗(d|00〉−c|11〉)	Z⊗I	ZX	ZX⊗X
	|−〉	(m|00〉−n|11〉)⊗(b|0〉−a|1〉)⊗(d|00〉+c|11〉)	Z⊗I	ZX	X⊗X
$|\phi_{3}\rangle ⊗|\psi_{4}\rangle$	|+〉	(m|11〉+n|00〉)⊗(b|0〉−a|1〉)⊗(d|00〉−c|11〉)	X⊗X	ZX	ZX⊗X
	|−〉	(m|11〉+n|00〉)⊗(b|0〉−a|1〉)⊗(d|00〉+c|11〉)	X⊗X	ZX	X⊗X
$|\phi_{4}\rangle ⊗|\psi_{4}\rangle$	|+〉	(m|11〉−n|00〉)⊗(b|0〉−a|1〉)⊗(d|00〉−c|11〉)	ZX⊗X	ZX	ZX⊗X
	|−〉	(m|11〉−n|00〉)⊗(b|0〉−a|1〉)⊗(d|00〉+c|11〉)	ZX⊗X	ZX	X⊗X

**Table A2:** The environment required for the experiment and the corresponding version.

Environment	Edition
Programming Language	Python 3.7
Programming IDE	VS Code
Experimental Platform	IBM Quantum Lab
Quantum Simulator	ibmq-qasm-simulator
